# Antibiotic-resistance in medically important bacteria isolated from commercial herbal medicines in Africa from 2000 to 2021: a systematic review and meta-analysis

**DOI:** 10.1186/s13756-022-01054-6

**Published:** 2022-01-21

**Authors:** Abdul Walusansa, Savina Asiimwe, Jesca. L. Nakavuma, Jamilu. E. Ssenku, Esther Katuura, Hussein. M. Kafeero, Dickson Aruhomukama, Alice Nabatanzi, Godwin Anywar, Arthur K. Tugume, Esezah K. Kakudidi

**Affiliations:** 1 Department of Plant Sciences, Microbiology and Biotechnology, School of Biosciences, Makerere University, Kampala, Uganda; 2 Department of Medical Microbiology, Faculty of Health Sciences, Islamic University in Uganda, P. O. Box 2555, Kampala, Uganda; 3 College of Veterinary Medicine, Animal Resources and Biosecurity, Makerere University, P. O. Box 7062, Kampala, Uganda; 4 Department of Medical Microbiology and Immunology, Faculty of Health Sciences, Busitema University, Mbale, Uganda; 5 Department of Immunology and Molecular Biology, Faculty of Health Sciences, Makerere University, Kampala, Uganda

**Keywords:** Africa, Antimicrobial resistance, Bacterial contamination, Herbal medicine, Meta-analysis, Systematic review

## Abstract

**Background:**

Antimicrobial resistance is swiftly increasing all over the world. In Africa, it manifests more in pathogenic bacteria in form of antibiotic resistance (ABR). On this continent, bacterial contamination of commonly used herbal medicine (HM) is on the increase, but information about antimicrobial resistance in these contaminants is limited due to fragmented studies. Here, we analyzed research that characterized ABR in pathogenic bacteria isolated from HM in Africa since 2000; to generate a comprehensive understanding of the drug-resistant bacterial contamination burden in this region.

**Methods:**

The study was conducted according to standards of the Preferred Reporting Items for Systematic Reviews and Meta-analyses (PRISMA). We searched for articles from 12 databases. These were: PubMed, Science Direct, Scifinder scholar, Google scholar, HerbMed, Medline, EMBASE, Cochrane Library, International Pharmaceutical Abstracts, Commonwealth Agricultural Bureau Abstracts, African Journal Online, and Biological Abstracts. Prevalence and ABR traits of bacterial isolates, Cochran’s Q test, and the I^2^ statistic for heterogeneity were evaluated using MedCalcs software. A random-effects model was used to determine the pooled prevalence of ABR traits. The potential sources of heterogeneity were examined through sensitivity analysis, subgroup analysis, and meta-regression at a 95% level of significance.

**Findings:**

Eighteen studies met our inclusion criteria. The pooled prevalence of bacterial resistance to at least one conventional drug was 86.51% (95% CI = 61.247–99.357%). The studies were highly heterogeneous (*I*^2^ = 99.17%; *p* < 0.0001), with no evidence of publication bias. The most prevalent multidrug-resistant species was *Escherichia coli* (24.0%). The most highly resisted drug was Ceftazidime with a pooled prevalence of 95.10% (95% CI = 78.51–99.87%), while the drug-class was 3^rd^ generation cephalosporins; 91.64% (95% CI = 78.64–96.73%). None of the eligible studies tested isolates for Carbapenem resistance. Extended Spectrum β-lactamase genes were detected in 89 (37.2%) isolates, mostly *Salmonella* spp., *Proteus vulgaris*, and *K. pneumonia*. Resistance plasmids were found in 6 (5.8%) isolates; the heaviest plasmid weighed 23,130 Kilobases, and *Proteus vulgaris* harbored the majority (n = 5; 83.3%).

**Conclusions:**

Herbal medicines in Africa harbor bacterial contaminants which are highly resistant to conventional medicines. This points to a potential treatment failure when these contaminants are involved in diseases causation. More research on this subject is recommended, to fill the evidence gaps and support the formation of collaborative quality control mechanisms for the herbal medicine industry in Africa.

## Background

Antimicrobial resistance (AMR), is the ability of bacteria, viruses, fungi, and parasites to evade the medicinal activity of drugs to which they were once susceptible [1]. The AMR makes effective treatment difficult or impossible. The major consequences include; increased cost of health care, prolonged hospital stays, and escalation of morbidity and mortality [2]. Currently, AMR causes over 700,000 global annual deaths, and the burden is intensifying rapidly worldwide [3, 4]. The widespread use of antibiotics, more so under inappropriate prescription in Africa, makes antibiotic resistance (ABR) a predominant form of AMR [5]; and the rates of antibiotic resistance are already alarming in some bacteria, such as *Escherichia coli*, *Klebsiella pneumonia*, *Salmonella spp.*, *Acinetobacter baumannii*, and *Staphylococcus aureus* [4–12]. These pathogens may spread from infected humans and/or animals to the environmental reservoirs such as; plants, water, soil, and subsequently to the rest of the community in a continuous cycle [the one health concept] [13]. Though often neglected, the potential role of herbal medicine (HM), given its widespread use, is intensifying the burden of ABR and necessitates substantive redress [14].

Globally, the prevalence of HM use ranges between 50 and 95%, and it is projected to rise with a compound growth rate of 5.5% by the year 2027 [15, 16]. In sub-Saharan Africa, the rate of HM consumption is reported to be over 60% [17], and it is used to treat and/or prevent health complications that range from instant emergencies, such as snakebite envenomation, to chronic conditions like; cancer, diabetes, HIV/AIDS-related symptoms, infertility, ulcers, and kidney diseases among others [17–21]. Bacterial pathogens have been documented as major contaminants that may be disseminated in herbal medicines and related products [22, 23]. Consequently, research and case reports concerning the profiles of bacteria that contaminate HM have been published, and their findings have been examined in some scientific reviews [22, 24–28]. In Europe, recent systematic reviews documented *Salmonella spp*., *Escherichia coli, Clostridium perfringens,* and *Listeria monocytogenes* among the frequently reported contaminants of commercial HM [25, 28]. Bacterial diseases associated with the consumption of contaminated HM were also diagnosed in the population in Europe [25]. However, the drug resistance traits of these pathogenic contaminants were not elucidated. In South Africa, bacteria, such as; *Pseudomonas spp.*, *Salmonella spp*., *Acinetobacter baumannii*, *Klebsiella pneumoniae*, and *Staphylococcus aureus,* that are capable of impairing human health have been reported in commercial medicinal herbal products [29]*.* The *Staphylococcus aureus* exhibited resistance to some of the conventional antibacterial drugs tested such as Methicillin and Vancomycin. Additionally, bacterial toxins such as *Bacillus cereus* diarrheal toxin have also been reported [29].

The contamination of HM with bacteria (which may be drug-resistant), raises concerns related to the community spread of antibiotic resistance. Though some researchers have continued to examine the loads and diversity of bacteria that contaminate HM in Africa, there is a shortage of comprehensive, continent-wide, scientific evidence, to explain the drug resistance patterns, and the genetic basis of resistance in these bacteria. This hinders the design of concerted herbal safety interventions and AMR stewardship programs on the continent. Therefore, this systematic review examined the original research articles related to drug resistance phenotypes, and the genes mediating this resistance, in potentially pathogenic bacteria that have been reported to contaminate HM in Africa in the past two decades. The rationale was to inform the design of collaborative approaches to support African countries in combating antimicrobial resistance.

## Materials and methods

### Study area

This meta-analysis included all the 54 countries that are located in the African region, as described by United Nations [30].

### Search strategy

Relevant key terms were used (initially used singly and later combined via linking words like, ‘‘with’’, ‘‘and’’, ‘‘or’’, ‘‘plus’’), to search twelve electronic databases (Table 1), for published articles relating to drug-resistant bacterial contamination of herbal medicine in the 54 countries [30]. This search included articles published from January 2000 to May 2021 and yielded 6,396 results.

**Table 1:** Databases searched, and the search terms used to identify publications on drug-resistant bacterial contamination of herbal medicines in Africa since 2000

Databases searched	Search terms
PubMed, Science Direct, Scifinder Scholar, Google scholar, HerbMed, Medline, EMBASE, Cochrane Library, International Pharmaceutical Abstracts, Commonwealth Agricultural Bureau Abstracts, Biological Abstracts, African Journal Online (AJOL)	Herbal medicine, Indigenous traditional medicine, Microbial herbal contamination, bacterial herbal contamination, Herbal medicine safety, Herbal medicine risks, Bacteria, Bacterial drug resistance, Bacterial drug resistance genes, Africa, Uganda, Nigeria, Ethiopia, Egypt, Democratic Republic of Congo, Tanzania, South Africa, Kenya, Algeria, Sudan, Morocco, Angola, Mozambique, Ghana, Madagascar, Cameroon, Cote d'Ivoire, Niger, Burkina Faso, Mali, Malawi, Zambia, Senegal, Chad, Somalia, Zimbabwe, Guinea, Rwanda, Benin, Burundi, Tunisia, South Sudan, Togo, Sierra Leon, Libya, Congo, Liberia, Central African Republic, Mauritania, Eritrea, Namibia, Gambia, Botswana, Gabon, Lesotho, Guinea-Bissau, Equatorial Guinea, Mauritius, Eswatini, Djibouti, Comoros, Cabo Verde, Sao Tome and Principe, Seychelles

### Selection criteria

Initially, all published literature related to the contamination of herbal medicine with drug-resistant bacteria in Africa was collected irrespective of the quality, research design used, and the attributes of the herbal samples studied, such as formulation, diseases treated, dosage, storage, precautions, and adverse effects. The final selection and inclusion of the publications were done using standardized protocols [31]. Studies that were included met the following conditions: they must have been full-text articles published in the English language, in peer-reviewed journals, between 2000 and 2021; and must have performed isolation, identification, and phenotypic and/or genotypic drug-resistance profiling of bacterial contaminants in commercial HM in African countries. The exclusion was based on: research conducted outside Africa, research investigating other microbial contaminants and adulterants, review articles, and research published after January 2000 and before May 2021.

### Review process

#### Data extraction from the journal articles

Six reviewers (AW, HMK, SA, EK, AN, GA), extracted data independently from the 18 eligible articles. Each researcher individually entered the data in spreadsheets, capturing these attributes: first author, year of publication, country, formulation, disease (s) treated, sample size, sampling techniques, mode of administration, potentially pathogenic bacterial species isolated, number of samples contaminated with bacteria, number of samples contaminated with drug-resistant bacteria, drug resistance phenotypes reported, drug resistance genes detected, and resistance plasmids. The reviewers compared their records every week to remove any duplicates and reconcile their data through a consensus.

#### Quality assessment

Quality assessment for the eligible studies was independently performed by four reviewers (AW, SA, JES, and DA), and a quality score ranging from 0 to 10 was awarded to each study. Quality scoring was done based on three dimensions namely; sample collection, comparability, outcome, and statistical analysis, as described in guidelines of the Newcastle–Ottawa scale [31]. Studies with a score of 9–10 were described as very good, 7–8 as good study, 5–6 as satisfactory study, and less than 5 as unsatisfactory. Consistency in quality assessment of the articles was supervised by two co-authors not involved in the scoring, i.e. (EKK and JLN).

#### Data analysis

The number of eligible studies and combined frequencies of potentially pathogenic bacterial species, drug resistance phenotypes, and drug resistance genes reported in the research articles, and proportions of herbal medicine samples contaminated with drug-resistant bacteria, were evaluated and presented using graphs and tables. A random-effects model was used to determine the pooled prevalence of drug-resistant bacteria, as well as their resistance traits, from the studies where heterogeneity was high; however, a fixed-effects model was used in cases where heterogeneity of the respective studies was low [32]. The results were presented using forest plots. Pooled prevalences were compared for association with different variables during the subgroup analysis and the P values at 95% CI were determined. Cochran's Q test and the I^2^ statistic were evaluated to examine the heterogeneity of the eligible studies for our meta-analysis. Publication bias was examined by constructing funnel plots. Sources of heterogeneity of the eligible studies were assessed by conducting sensitivity analysis, subgroup analysis, and meta-regression. All the analyses were performed using statistical software called MedCalcs (https://www.medcalc.org/), and *P* < 0.05 was considered significant in all cases. Five authors (AW, JES, DA, GA, AKT), were involved in the data analysis.

## Results

### Screening for eligible studies

**Fig. 1:**
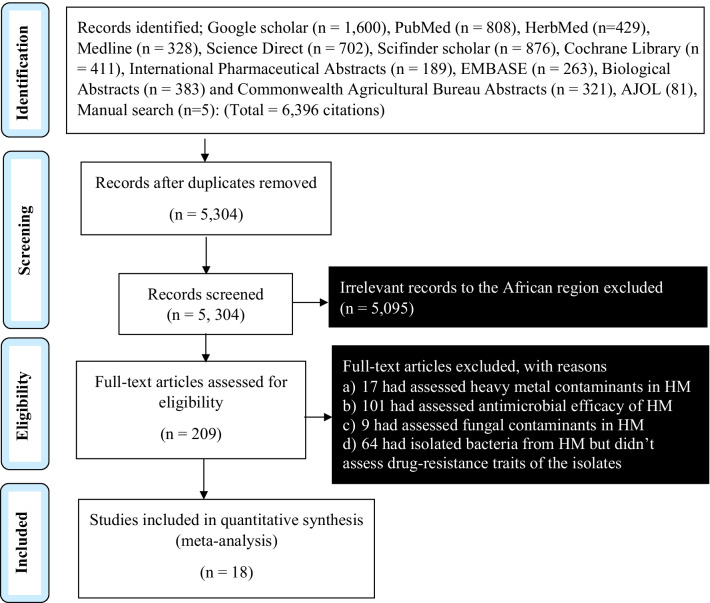
Flow chart for study eligibility screening of the research articles related to drug-resistant bacterial contamination of commercial herbal medicine in Africa, following PRISMA criterion

A standard search strategy, i.e., the Preferred Reporting Items for Systematic Reviews and Meta-Analyses (PRISMA), was used to screen for eligibility of the published articles concerning drug-resistant bacterial contamination of herbal medicines in Africa between 2000 and 2021 [33]. Eighteen research articles with a total sample size of 1111 met our inclusion criteria (Fig. 1).

**Fig. 2:**
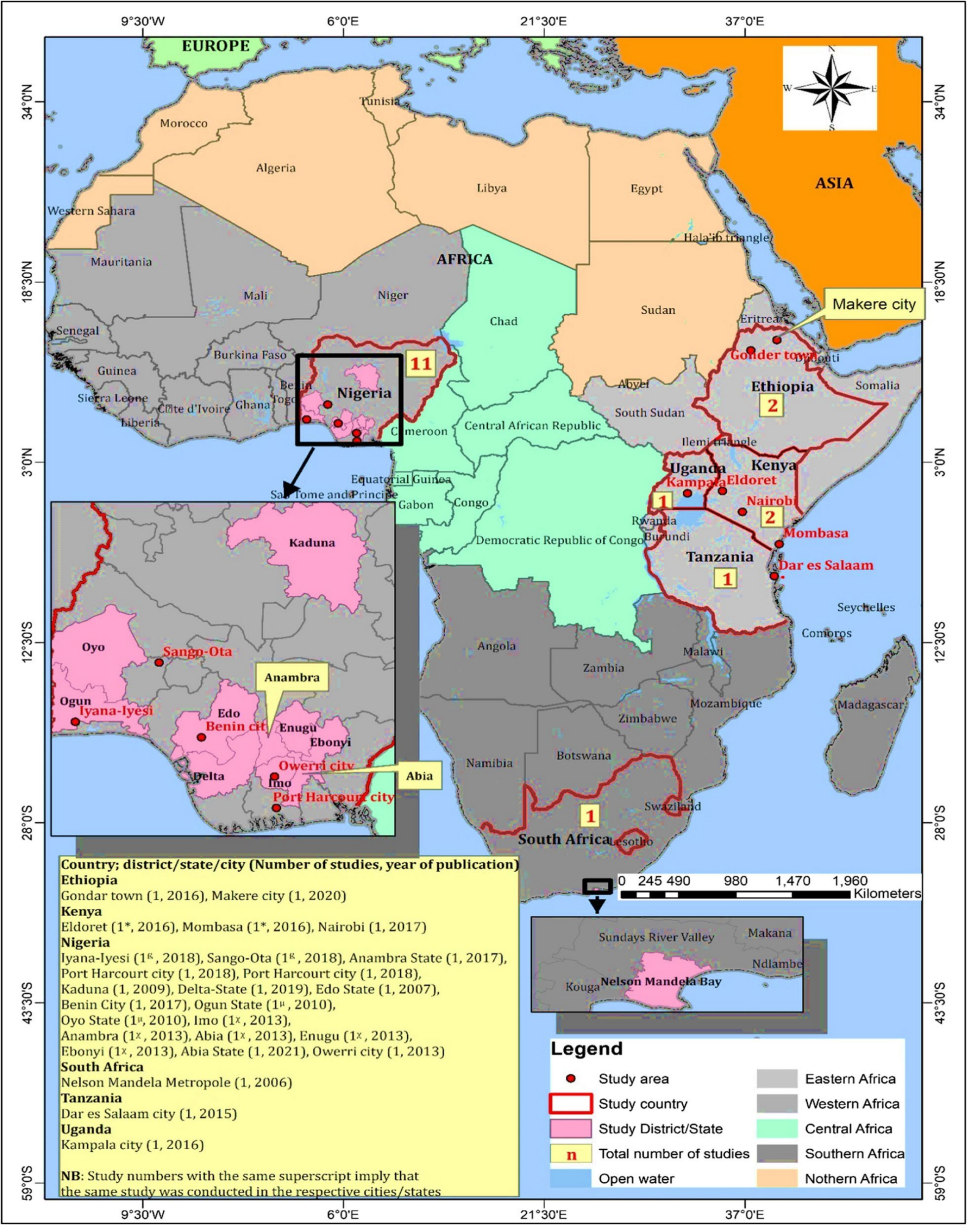
Distribution of representative countries that published research articles on drug resistance traits of medically important bacteria isolated from commercial herbal medicine in Africa from 2000 to 2021

The recent studies that suited our inclusion criteria were found in Uganda, Ethiopia, Nigeria, Tanzania, Kenya, and South Africa (Fig. 2).

### Characteristics of eligible studies on drug resistance traits of medically important bacteria isolated from commercial herbal medicines in Africa from 2000 to 2021

**Fig. 3:**
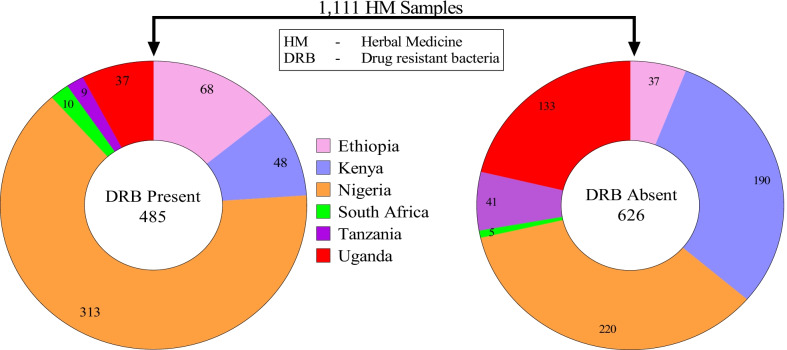
Distribution of commercial herbal medicines laden with drug-resistant bacterial contaminants in Africa from 2000 to 2021

**Fig. 4:**
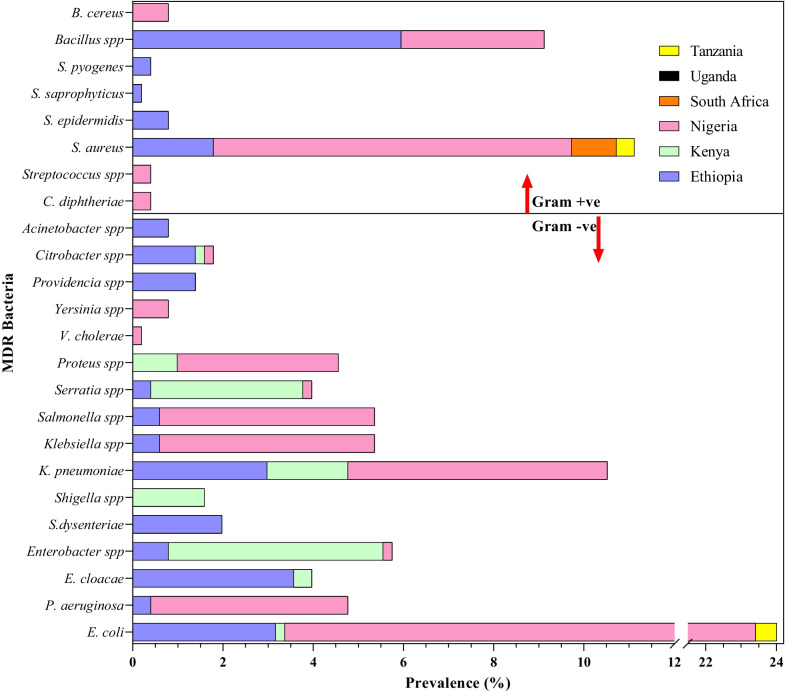
Spectrum of multidrug-resistant bacteria isolated from commercial herbal medicines in Africa from 2000 to 2021

The characteristics of eligible studies are summarized in Table 2, Figs. 3 and 4. A total of 18 eligible studies, with a sample size of 1111 were included in this Meta-Analysis. Nigeria had the greatest number of eligible studies (11, 61%), with a total sample size of 533, followed by Kenya and Ethiopia with (2, 11%) studies each and total sample sizes of 238, and 105 respectively. A study by Niyoshima, 2016 in Uganda, had the largest sample size of 170, while studies in Nigeria by; Osungunna et al. 2010, Omoruyi et al. 2017, and Braide et al. 2013 had the smallest sample size of 10 each. Most studies were published between the years 2011 and 2021 (14/18, 77.8%), with a total sample size of 909 (81.8%) compared to the studies published in 2000–2010 (4/18; 22.2%), with a total sample size of 201 (18.2%). All the eligible studies used conventional culture to isolate bacterial contaminants from HM and phenotypic methods (colony morphology, gram staining, and biochemicals), to characterize the isolates. These studies used Kirby-Bauer disk-diffusion methods to profile the drug resistance phenotypes of bacterial isolates, and one study (4.5%) additionally applied Polymerase Chain Reaction (PCR) to examine the resistance genotypes [14].

**Table 2:** Characteristics of studies on drug resistance traits of medically important bacteria isolated from commercial herbal medicine in Africa from 2000 to 2021 (N = 18)

First author, year	Country	Malady	Study design	Sampling technique	Sample size	SRB (N)	Total bacteria isolated	Bacteria Screened	Bacteria ROD [N, (%)]; Species	MDR Bacteria [N, (%)]; Species	Least potent drugs	MDR phenotypes/genotypes	Resistance Plasmids (N)	References
Abdela et al. 2016	Ethiopia	UTI, Mycoses, Cancer, Paralysis, Diarrhea, Malaria	CSD	Purposive	55	50	150	150	131, (87.3%); *E. coli, S. aureus, S. pyogenes, P. aeruginosa, Bacillus spp., E. cloacae, S. dysenteriae, K. pneumoniae, S. epidermidis, S. saprophyticus, Salmonella spp., Enterobacter spp., Klebsiella spp., Providencia spp., Citrobacter spp., Serratia spp., Acinetobacter spp.*	125, (83.4%); *Salmonella spp., Enterobacter spp., Klebsiella spp., Providencia spp., Citrobacter spp., Serratia spp., Acinetobacter spp., E. coli, S. aureus, S. pyogenes, P. aeruginosa, Bacillus spp., E. cloacae, S. dysenteriae, K. pneumoniae, S. epidermidis, S. saprophyticus*	Ampicillin*, AMC**	NT	NT	[34]
Kidus et al. 2020	Ethiopia	Jaundice, Constipation	CSD	Purposive	50	18	39	39	25, (64%); *E. coli*, *Bacillus spp.*, *Micrococcus spp.*	13, (33.3%); *Bacillus spp.*, *E. coli*	Ampicillin*, Rifampicin**	NT	NT	[35]
Keter et al. 2016	Kenya	Malaria, Typhoid, Hypertension, Pneumonia, allergies, STDs, Cancer, Diabetes, Impotence, Wounds, UTIs, HIV	Exp, Ept	Purposive	100	13	164	106	14; (13.2%); *S*. *intermedius, S. erwania, Proteus spp., Shigella spp., K. pneumonia, E. coli, Enterobacter spp., Citrobacter spp.*	3, (2.8%); *K. pneumoniae, Shigella spp., S. erwania, Enterobacter spp., Proteus spp., E. coli, Citrobacter spp.*	Cefepime*, Cefotaxime**	NT	NT	[36]
Korir et al. 2017	Kenya	Unspecified	Exp, Ept	Purposive	138	35	101	96	96, (100%); *E. cloacae, P. penneri, C. diversus, E. aerogenes, K. pneumoniae, E. cloacae*	2; (2%); *E. cloacae*	Ceftazidime*, Cefotaxime**	ESBL	NT	[14]
Oluwasegun et al. 2018	Nigeria	Unspecified	LSD	Purposive	32	32	41	24	24, (100%); *E. coli, V. chole, S. typhi, S. aureus*	*20,* (83%); *V. cholerae S. typhi, S. aureus,E. coli*	Ceftazidime*, Ciprofloxacin**	NT	NT	[37]
Archibong et al. 2017	Nigeria	Unspecified	CSD	Purposive	60	49	94	50	50, (100%); *Bacillus spp., Streptococcus spp., A. baumannii, E. coli*	*15, (30%); E. coli,Bacillus spp., Enterobacter spp., S. aureus*	Streptomycin*, AMC**	NT	NT	[38]
Stanley et al. 2018	Nigeria	Unspecified	CSD	Random	40	18	30	30	22, (73%); *E. coli, S. aureus, Klebsiella spp., P. aeruginosa*	*20,* (67%); *E. coli, S. aureus, Proteus spp., Klebsiella spp.*	Ceftazidime*, Ciprofloxacin**	NT	NT	[39]
Abba et al. 2009	Nigeria	Unspecified	CSD	Random	150	0	285	285	0	0	0	NT	NT	[40]
Ejukonemu et al. 2019	Nigeria	Typhoid, Malaria, Erectiledysfunction, general Infections, Rheumatism	CSD	Random	25	25	53	53	53, (100%); *Klebsiella spp.,E. coli, Salmonella spp., S. aureus, P. mirabilis, Enterobacter spp.*	53, (100%); *Klebsiella spp., Salmonella spp., E. coli, S. aureus, Proteus spp.*	Ampicillin*, Ceporex**	NT	NT	[41]
Osungunna et al. 2010	Nigeria	Annal fistula	CSD	Purposive	10	10	20	20	17, (85%); *P. aeruginosa, Klebsiella spp., S. aureus*	0	Nalidixic acid*, Ofloxacin**	NT	NT	[42]
Esimone et al. 2007	Nigeria	Unspecified	CSD	Random	26	11	75	75	75, 100%; *B. cereus, B. subtilis, E. coli, Streptococcus spp., S. epidermidis, K. pneumoniae, S. aureus, Serratia spp., Lactobacillus spp., Citrobacter spp., Listeria spp., P. aeruginosa*	75, 100%; *B. cereus, B. subtilis, E. coli,Streptococcus spp., S. epidermidis, K. pneumoniae, S. aureus, Serratia spp., Lactobacillus spp, Citrobacter spp., Listeria spp., P. aeruginosa*	Cefuroxime*, Nitrofurantoin**	NT	NT	[43]
Omoruyi et al. 2017	Nigeria	Unspecified	CSD	Random	10	10	10	6	6, (100%); *E. coli*, *Klebsiella spp.*, *Citrobacter spp.*, *Serratia spp.*	6; 100%; *E. coli, Klebsiella spp., Citrobacter spp., Serratia spp.*	Ceftazidime*, AMC**	ESBL	NT	[44]
Ujam et al. 2013	Nigeria	Unspecified	CSD	Random	20	18	49	45	45, 100%; E. coli, *P. aeruginosa, Staphylococcusspp., Salmonella spp., Streptococcus spp., Bacillus spp., Proteus spp., Yersinia spp., C. diphtheria*	45, (100%); *E. coli, P. aeruginosa S. aureus, Salmonella spp., Streptococcusspp, Bacillus spp., Proteus spp., Yersinia spp., C. diphtheria*	Cotrimoxazole*, Ampicillin**	NT	NT	[45]
Braide et al. 2013	Nigeria	Typhoid, STDs, Piles, Stomach aches, Diabetes, Headache,Skin infections, Toothache	CSD	Random	10	10	45	34	34, (100%); *S. aureus, E. faecalis, B. subtilis, C. diphtheriae, M. luteus*	0	Amoxicillin*, Chloramphenicol**	NT	NT	[46]
Nwankwo et al. 2021	Nigeria	Unspecified	CSD	Unspecified	150	130	315	274	274, (100%); *Salmonella* spp*, E. coli, P. vulgaris, Citrobacter freundii, P. aeruginosa, K. pneumonia,S. aureus, Streptococcus spp.*	98, (36%); *Salmonella spp., E. coli, Proteus spp., K. pneumoniae*	AMC*, Ciprofloxacin**	ESBL	6	[47]
Govender et al. 2006	South Africa	HIV/AIDS-related complications	CSD	Unspecified	15	10	32	20	16, (80%); *S. aureus, B. cereus*	5, (31%); *S. aureus*	Methicillin*, Vancomycin**	NT	NT	[29]
Kira 2015	Tanzania	Unspecified	CSD	Random	50	9	40	32	17, (43%); *S. aureus, E. coli*	5, (16%); *E. coli, S. aureus*	Vancomycin*, Cefotaxime**	NT	NT	[48]
Niyoshima 2016	Uganda	Unspecified	CSD	Random	170	37	69	60	60, (100%); *S. aureus*	0	Penicillin*	NT	NT	[49]

Key: CSD; Cross-sectional Design, Ept; Exploratory, Exp; Experimental, SRB; Samples with resistant bacteria, ROD; Resistant to at least one drug, MDR; Multidrug-Resistant, STDs; Sexually Transmitted Disease, UTIs; Urinary Tract Infections, HIV; Human Immunodeficiency Virus, AMC; Amoxicillin + Clavulanate, NT; Not Tested, Ref; Reference,

P. *aeruginosa* = *Pseudomonas aeruginosa, E. coli* = *Escherichia coli, S. aureus* = *Staphylococcus aureus; K. pneumoniae; Klebsiella pneumoniae,* *; Least potent drug, **; Second least potent drug, LSD; Longitudinal Study Design, ESBL; Extended Spectrum Beta Lactamases, MW; Molecular Weight, *kb*; kilobases

The distribution of samples that were found to possess drug-resistant bacterial contaminants (Fig. 3).

**Fig. 5:**
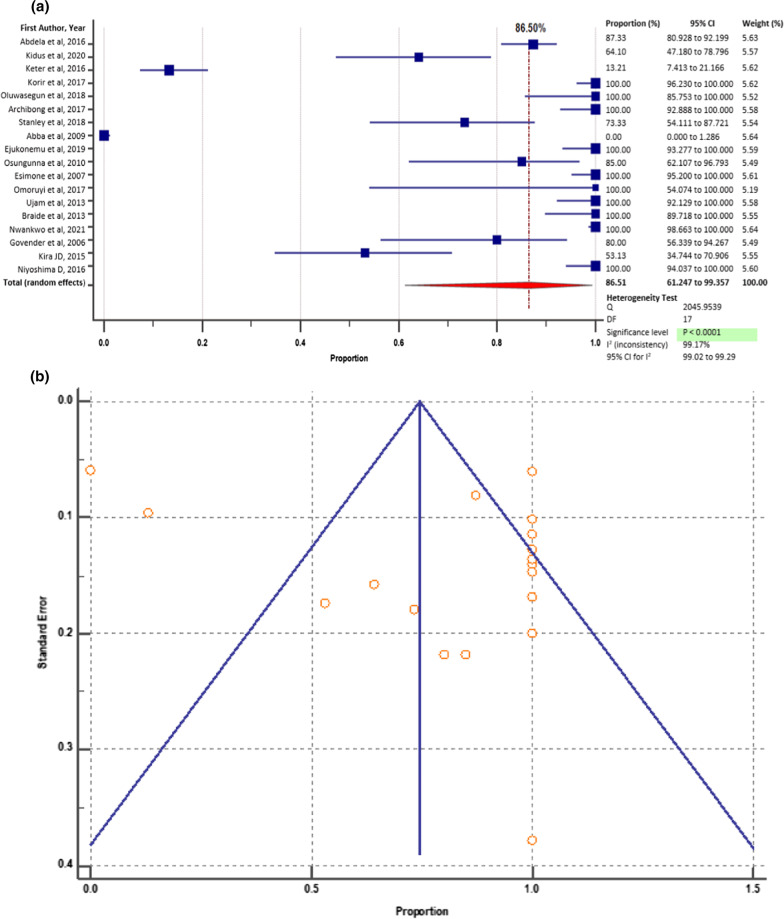
**a** Pooled prevalence of resistance to at least one conventional drug in bacteria isolated from herbal medicines in Africa from 2000 to 2021; **b** Bias assessment plot of studies that reported the drug-resistant bacterial contaminants

Overall, 1612 bacterial strains of potential medical importance were isolated from 1111 herbal medicine samples. Of these, 1,399 isolates were screened for drug resistance traits. A total of 1210 (86.5%), isolates exhibited resistance to at least one antibacterial drug. The prevalence of such bacteria among the countries where eligible studies existed varied widely. A study conducted by Abba et al. 2009 in Nigeria reported the least prevalence of bacteria that were resistant to at least one conventional drug: 0% (95% CI = 0.00–5.96%) [40]. In their study, all the 285 isolates were found to be sensitive to the drugs tested i.e., Amoxicillin, Erythromycin, Ampicillin, Ofloxacin, Cefaclor, Streptomycin, Chloramphenicol, and Tetracycline. The highest prevalence of bacteria that were resistant to at least one antibacterial agent was 100%, and this was reported by 9 (50%) of the eligible studies, found in Kenya and Nigeria [14, 37, 38, 41, 43–47], (Fig. 5a). The most potent antibiotic as reported by 6 (33.30%) of the eligible studies was Gentamicin. Multidrug resistance (MDR) [Resistance of a strain to at least three drugs that belong to different drug classes] were reported in 504 (36.0%) of the 1, 399 isolates screened. Nigeria reported the highest number of MDR pathogens (290/504, 57.80%), while Uganda reported the least (0/504, 0.00%). *Escherichia coli* was the most frequently reported MDR species (121/504, 24.01%), followed by *Staphylococcus aureus* (56/502, 11.1%) as shown in Fig. 4.

### The pooled prevalence of bacterial contaminants that were resistant to at least one conventional drug in the African countries between 2000 and 2021

The overall pooled prevalence of bacteria that were resistant to at least one drug, among the herbal medicines in Africa from 2000 to 2021 was 86.51% (95% CI = 61.247–99.357%), with heterogeneity (*I*^2^) of 99.17% (*p* < 0.0001) (Fig. 5a). We constructed a funnel plot to gauge publication bias. Though the eligible studies were highly heterogeneous (*p* < 0.0001), the funnel plot exhibited symmetrical spread in terms of relative weight and effect size, therefore demonstrating no evidence of publication bias (Fig. 5b).

### Meta-analysis of sub-groups

Since the eligible studies were highly heterogeneous, we clustered the analysis into seven categories. The clustering was based on (a): prevalence of multi-drug resistant bacteria per (i) country, (ii) year of publication, (iii) disease treated; (b) Least potent antibacterial drugs; (c) Least potent drug classes; (d) Drug resistance genes detected; and (e) Resistance plasmids detected (Table 2). Nations with only one eligible study, viz; Uganda, Tanzania, and South Africa were left out during meta-analysis of the sub-group “country of study”. Similarly, diseases treated, and the least potent drugs, that were reported by a single study were not included during the meta-analysis of the respective sub-groups. Only one type of antibiotic resistance genes was reported, viz: Extended Spectrum β-Lactamases (ESBL) [14], and a single study in Nigeria, by Nwankwo et al. [47], reported the presence of resistance plasmids; thus the sub-groups, “drug resistance genes detected”; and “resistance plasmids detected” were considered unsuitable for meta-analysis.

In most of the categories considered for sub-group analysis, heterogeneity (*I*^2^) declined below the value (*I*^2^ = 97.6%, *p* ≤ 0.0001) reported in the overall meta-analysis of bacterial resistance to at least one drug (Fig. 5a); except for MDR prevalence in the years 2011 to 2021 (98.16%, *p* ≤ 0.0001), erectile dysfunction (*I*^2^ = 98.09%; *p* ≤ 0.0001), Typhoid (98.83%, *p* ≤ 0.0001), diabetes (98.57%, *p* ≤ 0.0001), and the herbal drugs for which the published studies (n = 11), did not specify the type diseases that were claimed to treat (97.67%, *p* ≤ 0.0001) (Table 2).

At the country level, the highest and lowest prevalence of multi-drug resistance phenotypes in bacterial contaminants of herbal medicines were reported in Ethiopia; 60.18% (95% CI = 13.15–97.27%), and Kenya; 25.53% (95% CI = 3.70 to 90.23%) respectively (Table 2). There was no evidence of publication bias in the countries (Ethiopia, Kenya, and Nigeria) that were considered for this sub-group. The pooled prevalence of MDR bacteria in Ethiopia was significantly higher than in Kenya (*p* < 0.0001) and in Nigeria (*p* = 0.0364).

About the variation of multi-drug resistant bacterial strains per year of publication, the period between 2011 and 2021 (N = 14) registered the highest pooled prevalence of MDR bacteria, 50.02% (95% CI = 27.20–72.83%), as compared to 11.80% (95% CI = 7.85–41.54%) registered between 2000 to 2010 (n = 4). The prevalences were significantly different (*p* < 0.0001), in the two study periods (Table 2).

About the disease categories, the herbal drugs suggested for the management of erectile dysfunction harbored the highest pooled prevalence of MDR bacterial contaminants: 86.78% (95% CI = 32.51–95.25%), and this was significantly different from the prevalence in the herbal drugs used to manage the rest of the diseases except malaria (*p* = 0.7301). The herbal drugs proposed for treating diabetes contained the lowest MDR prevalence; 22.54% (8.88 to 92.43%).

Ceftazidime (n = 3), was the least potent antibacterial drug. The pooled prevalence of resistance to Ceftazidime was 95.10% (95% CI = 78.51 to 99.87%), followed by Ampicillin at 81.15% (CI = 47.61 to 99.21%). The pooled prevalence of resistance to these two drugs was significantly different (*p* = 0.0002). The least potent drug class was the 3rd generation Cephalosporins, with a pooled prevalence of 94.78% (73.65 to 99.39%). The pooled prevalence of 3rd generation Cephalosporin resistance was not significantly different from that of other drug classes, viz; Penicillins (*p* = 0.0677) and all β-lactam drugs combined (*p* = 0.3123) (Tables 2, 3). The anti-bacterial drugs and drug classes that were reported as least potent in a single study, hence excluded from the meta-analysis are shown in Table 4. In these studies, resistance to Augmentin (Amoxicillin/clavulanic) manifested in the greatest number of isolates (n = 154; 56.2%), and this was significantly different from resistance to other drugs except Cefepime (χ^2^ = 0.937; *p* = 0.3331), Methicillin (χ^2^ = 3.360; *p* = 0.0668), and Vancomycin (χ^2^ = 38.197; *p* = 0.8076). Among the drug classes, there was high resistance to β-lactam + β-lactamase inhibitors (n = 274; 100.0%), and minimal resistance to Glycopeptide drugs (n = 17; 53.1%). The resistance to Glycopeptides was significantly lower than the rest of the drug-classes except 4^th^ generation Cephalosporins (χ^2^ = 0.575; *p* = 0.4481) and Quinolones (χ^2^ = 3.928; *p* = 0.0475) (Table 3).

**Table 3:** Sub-group analysis of the pooled prevalence of multidrug resistance, and the least potent drugs among bacterial contaminants of herbal medicines in Africa from 2000 to 2021

Variable	Analysis				
	Number of studies	Prevalence % (95% CI)	*P* value	I^2^ (%) (95% CI)	*P* het
**MDR bacteria per country of study**					
Ethiopia	2	60.18 (13.15 to 97.27) REF		97.16 (92.69 to 98.90)	< 0.0001
Kenya	2	25.53 (3.70 to 90.23)	< 0.0001	99.10 (98.25 to 99.54)	< 0.0001
Nigeria	11	49.35 (22.78 to 76.12)	0.0364	97.93 (97.24 to 98.44)	< 0.0001
**MDR bacteria per year of publication**					
2021 to 2011	14	50.02 (27.20 to 72.83) REF		98.16 (97.66 to 98.55)	< 0.0001
2010 to 2000	4	11.80 (7.85 to 41.54)	< 0.0001	95.08 (90.38 to 97.48)	< 0.0001
**MDR bacteria per disease treated**					
Erectile dysfunction	2	86.78 (32.51 to 95.25) REF		98.09 (95.58 to 99.18)	< 0.0001
HIV/AIDS complications	2	44.56 (13.58 to 78.15)	< 0.0001	89.11 (59.13 to 97.10)	0.0024
Urinary tract infections	2	72.99 (49.51 to 91.11)	0.0044	93.55 (49.51 to 91.11)	0.0001
Malaria	3	85.43 (59.59 to 99.11)	**0.7301**	96.23 (92.02 to 98.22)	< 0.0001
Cancer	2	72.99 (49.51 to 91.11)	0.0044	93.55 (79.10 to 98.01)	0.0001
Typhoid	3	54.50 (2.07 to 99.71)	< 0.0001	98.83 (98.03 to 99.31)	< 0.0001
Diabetes	2	22.54 (8.88 to 92.43)	< 0.0001	98.57 (96.91 to 99.34)	< 0.0001
Unspecified diseases	11	39.19 (17.32 to 63.65)	< 0.0001	97.67 (96.86 to 98.26)	< 0.0001
**Least potent drugs**					
Ceftazidime	3	95.10 (78.51 to 99.87) REF		87.48 (70.14 to 94.75)	< 0.0001
Ampicillin	3	81.15 (47.61 to 99.21)	0.0002	96.29 (92.17 to 98.24)	< 0.0001
**Least potent drug classes**					
3rd Generation cephalosporins	4	94.78 (73.65 to 99.39) REF		91.64 (78.64 to 96.73)	< 0.0001
Penicillins	6	89.57 (69.78 to 99.43)	**0.0677**	95.35 (92.21 to 97.22)	< 0.0001
All β-lactam drugs	13	92.45 (81.59 to 98.73)	**0.3123**	95.99 (94.47 to 97.09)	< 0.0001

Bolded *P*-values are not significant

MDR = Multi-Drug Resistance, ESBL = Extended Spectrum β-Lactamase, CI = Confidence Interval, het = Heterogeneity, HIV = Human Immunodeficiency Virus, AIDS = acquired immunodeficiency syndrome, REF = Reference value

**Table 4:** Antibacterial drugs and drug classes that were reported by single studies, to be the least potent among bacterial contaminants of herbal medicines in Africa, 2000–2021

Drug	Number studies	Isolates screened (N)	Resistant isolates N (%)	χ^2^	*P*-value
Augmentin	1	274	154 (56.2)		
Cefepime	1	106	67 (63.2)	0.937	**0.3331**
Streptomycin	1	50	50 (100.0)	32.558	< 0.0001
Cefuroxime	1	75	75 (100.0)	46.363	< 0.0001
Nalidixic acid	1	20	17 (85.0)	5.221	0.0223
Co-trimoxazole	1	45	45 (100.0)	29.667	< 0.0001
Amoxicillin	1	34	34 (100.0)	23.101	< 0.0001
Methicillin	1	20	16 (80.0)	3.360	**0.0668**
Vancomycin	1	32	17 (53.1)	0.0593	**0.8076**
Penicillin	1	60	60 (100.0)	38.197	< 0.0001
**Drug class**					
Glycopeptides	1	32	17 (53.1)		
2^nd^ generation Cephalosporins	1	75	75 (100.0)	38.094	< 0.0001
4^th^ generation Cephalosporins	1	106	67 (63.2)	0.575	**0.4481**
Aminoglycosides	1	50	50 (100.0)	26.220	< 0.0001
Quinolones	1	20	17 (85.0)	3.928	**0.0475**
Sulfonamides	1	45	45 (100.0)	23.829	< 0.0001
β-lactam + β-lactamase inhibitor	1	274	274 (100.0)	131.672	< 0.0001

Bolded *P*-values are not significant

χ^2^ = Chi-square

### Drug resistance enzymes, genes, and plasmids

Two studies (10%), studies screened the bacterial contaminants for MDR enzymes phenotypically [14, 47], while one study screened for MDR genes [44]. The former aimed at the detection of Extended Spectrum Beta-lactamase (ESBL) enzymes, while the latter screened for some of the genes that encode ESBL. Among the isolates screened for MDR enzymes and/or genes, 89 (37.2%) were ESBL positive, and these included; *Salmonella spp.*, *Proteus vulgaris*, *Klebsiella pneumoniae,* and *E. coli* among others (Table 5). About the resistance genes, *bla*_*CTX-M*_* -*ESBL and *bla*_*CMY*_*-*ESBL were confirmed in bacteria such as *K. pneumoniae,* and *E. cloacae* among others, in Nigeria [44]*.* Consequently, Nwankwo et al. 2021 screened for the presence of drug-resistance plasmids in 103 bacterial isolates in Nigeria; of these, 6 (5.8%) isolates were found to possess the targeted plasmids that were linked to the observed drug resistance phenotypes [47]. *P. vulgaris* possessed the highest number of plasmids (N = 5; 83.3%), with the largest possessing a molecular weight of 23,130 Kb, as shown in Table 1.

**Table 5:** Drug-resistance genes and plasmids identified in bacteria isolated from commercial herbal medicines in Africa from 2000 to 2021

Isolates screened (N)	MDR Phenotypes/genotypes	Isolates with MDR phenotypes/genotypes	
		N, (%)	Species
**(a) Multi-drug resistance phenotypes and/or genotypes**			
06	ESBL	1, (17.0%)	*K. pneumoniae*
98	ESBL	13, (13.3%)	*Salmonella spp*.
13	ESBL	4, (30.8%)	*E. coli*
13	ESBL	4, (30.8%)	*P. vulgaris*
13	ESBL	3, (23.1%)	*K. pneumoniae*
96	*bla*_*CTX-M*_* -*ESBL	33, (34.4%)	*P. penneri, K. pneumoniae, C. diversus, E. cloacae, M. morganii*
	*bla*_*CMY*_*-*ESBL	31, (32.3%)	*E. cloacae, E. aerogenes, K. pneumoniae*
Σ = 239		Σ = 89 (37.2%)	

Isolates tested (N)	Resistance plasmids detected		Isolates with drug resistance plasmids	
	Number	MW(*Kb*)	N, (%)	Species
**(b) Resistance plasmids**				
16	1	9416	1 (6.3%)	*Salmonella spp.*
11	2	23,130	2 (18.2%)	*P. vulgaris*
	1	2322	1 (9.1%)	
	1	9416	1 (9.1%)	
	1	2322	1 (9.1%)	
47	0	Nil	0 (0.0%)	*Escherichia coli*
29	0	Nil	0 (0.0%)	*Klebsiella pneumoniae*
Σ = 103	Σ = 06		Σ = 06 (5.8%)	

ESBL = Extended Spectrum *β*-lactamase enzymes, MW = Molecular Weight, *Kb* = kilobases

### Meta-regression

**Fig. 6:**
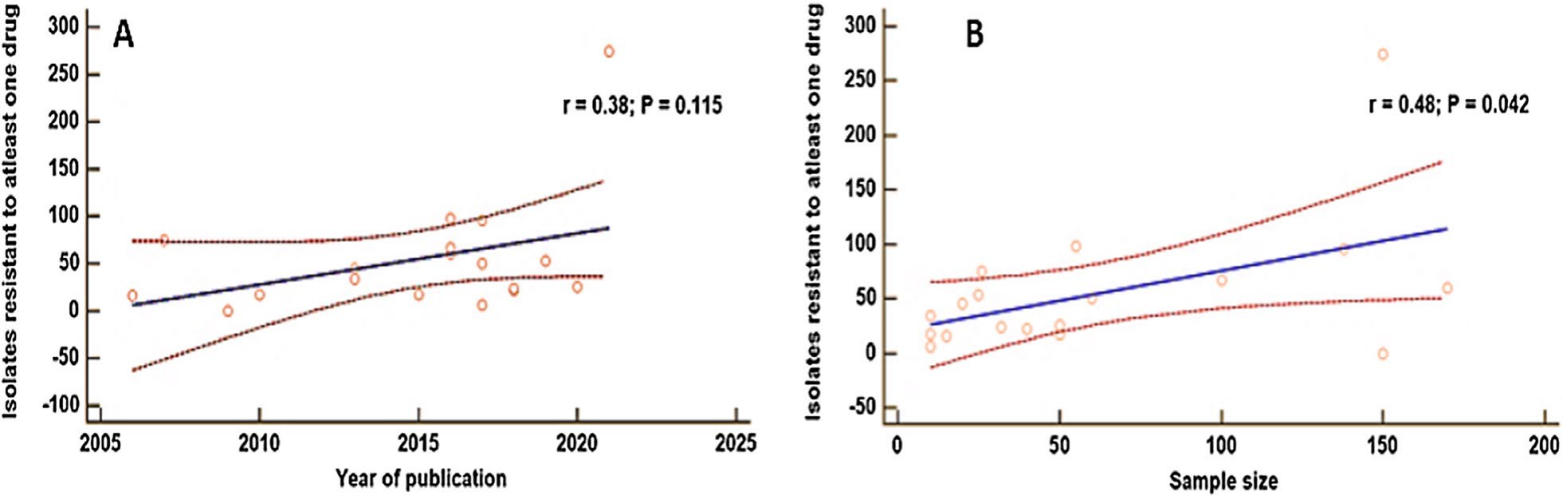
Meta-regression analysis by the prevalence of bacterial resistance to at least one drug and year of publication (**A**), as well as the sample size (**B**), of the herbal medicines sold in Africa from 2000 to 2021

Meta-regression analysis was performed to examine the continuous variables of bacterial resistance to at least one contemporary drug, and year of publication, as well as the sample size (*p* > 0.05). The results showed that the years of publication of the eligible studies were not significantly associated with the prevalence of bacterial resistance to at least one drug (*p* = 0.115) (Fig. 6a); however, the sample size was significantly associated with the prevalence of bacterial contaminants that were resistant to at least one modern antibacterial agent (*p* = 0.042) (Fig. 6b).

### Sensitivity analysis

**Fig. 7:**
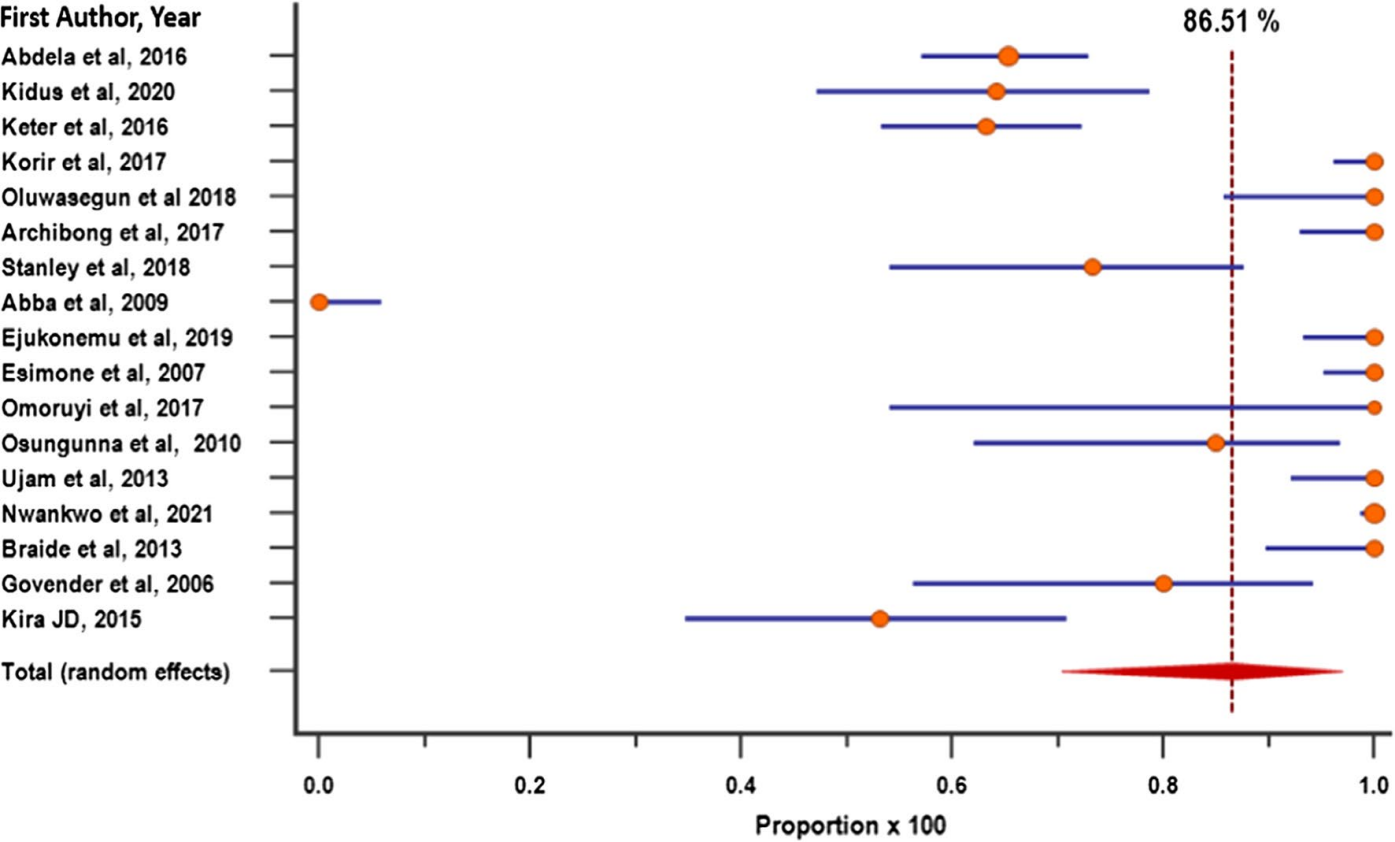
Forest plot showing sensitivity analysis of the prevalence of bacterial contaminants that were resistant to at least one drug

Sensitivity analysis involved the removal of one study which had the largest sample size [49]. Results show that there was a slight decline in the pooled prevalence of drug-resistant bacteria from the original 87.7% (95%CI = 72.82–97.18%) (Fig. 5a), to 86.51% (95% CI = 70.401–96.898) (Fig. 7), with heterogeneity (*I*^2^); 97.69% and *p* < 0.0001.

## Discussion

A total of eighteen original scientific studies that investigated the drug-resistance burden in bacteria recovered from commercial HM in Africa and published the findings online from 2000 to 2021 qualified for inclusion in this meta-analysis. It would be interesting to compare these findings with the number of studies reported earlier in other continents, however, systematic reviews and/or meta-analyses on this subject outside Africa remain scarce. In the current meta-analysis, the eighteen eligible studies were found in Uganda, Kenya, South Africa, Ethiopia, Tanzania, and Nigeria. Though some more studies related to bacterial contamination of herbal medicine were barely available in those six countries, plus a few other African states such as Malawi, Sudan, Cameroon, and Benin, the studies did not examine the drug-resistance traits of the isolated bacteria [23, 40, 50–61]. This highlights the need for more adequate research on this subject, in light of the escalating burden of antibacterial resistance in the African region [4–8]. The total sample size from the 18 studies was 1,111; from which 1,612 bacterial strains were isolated. Out of these bacteria, 1399 isolates were screened for drug sensitivity, and of these, 1210 exhibited resistance to at least one of the conventional antibacterial agents tested. Therefore, the pooled prevalence of resistance to at least one drug was 86.51% (95% CI = 61.247–99.357%); and the studies were highly heterogeneous (*I*^2^ = 99.17%; *p* < 0.0001), with no demonstratable evidence of publication bias.

In 10 (55.6%), of the eighteen eligible studies, the prevalence of resistance to at least one conventional drug was higher than the overall pooled prevalence (86.51%), with the highest, (100%), being reported by some studies in Kenya, Nigeria and Uganda (14,37,41,49). Overall, multi-drug resistance (MDR) phenotypes were reported in 504 (36.0%) of the 1399 isolates screened. Nigeria reported the highest prevalence of MDR pathogens, 57.80% (n = 290), with *Escherichia coli* being the most prevalent, while Uganda reported the lowest, 0.00% (n = 0). The low carriage of MDR in Uganda, as observed, remains elusive because only one eligible study was available for inclusion in this meta-analysis [49]. The eligible study aimed at isolating only one species (*S. aureus*), and the isolates were tested against a small number of antibiotics (Gentamicin, Chloramphenicol, Ampicillin, Penicillin, Tetracycline); besides the study did not attempt to examine the MDR traits [49].

The findings show that in the African region, *Escherichia coli* was the most frequently reported multidrug-resistant bacterial contaminant of herbal medicine, with a pooled prevalence of 24.01% (n = 121), followed by *Staphylococcus aureus* at 11.1% (n = 56); Others included; *Salmonella *spp., *Bacillus *spp., *Shigella *spp. and *Klebsiella pneumoniae* (Fig. 4). All these bacteria, except *Bacillus spp.*, exist on the global list of resistant pathogens which the World Health Organization has identified, that need priority in research, discovery, and development of new antibiotics [62, 63]. The predominance of coliform bacteria such as *E. coli* is suggestive of the highly compromised hygiene associated with fecal contamination of plants, water, and other environmental resources [22]. Therefore, the presence of such microbes in the medicinal herbal end-products might partly be attributed to the potential inadequacy of quality control during harvesting, transporting, processing, and/or packaging; poor compliance to Good Manufacturing Practices (GMPs) and/or regulatory frameworks; illegal operation of the herbal medicine businesses leading to absolute lack of monitoring by the authorities. Further, the presence of drug-resistance traits in some bacteria, such as *K. pneumoniae*, and *E. coli* that are recovered from consumable products like herbal medicines, or humans and animals, has been reported to be indicative of the levels of antibiotic-resistant bacterial pollution in the environment [64, 65]. Effective mitigation of the community spread of drug-resistant bacteria, therefore, requires a one health approach. One health is a collaborative, transdisciplinary scheme that promotes the achievement of optimal health outcomes given the interconnections among humans, plants, animals, and their shared environment [66].

Some of the highly prevalent MDR bacteria revealed by this meta-analysis are in alignment with the MDR strains isolated from commercial herbal medicines elsewhere. For example, in some parts of Asia, a high prevalence of MDR *K. pneumoniae*, *S. aureus*, and *E. coli* contaminants in herbal medicines have been reported [67]*.* Such species are commonly implicated in the community spread of multi-drug resistance in many parts of Europe and North America; with some studies reporting *K. pneumoniae* that has evolved resistance to all the currently known antibiotics [68, 69]. This indicates that the species of bacteria that are disseminated by herbal medicines in Africa represent some of the world’s most critical bacterial conduits of AMR beyond the African region.

Besides concerns related to AMR spread, the high prevalence of MDR coliforms such as *E. coli* in herbal medicine is indicative of the gross absence of hygiene, associated with fecal contamination of the herbal medicines. The contaminants may come from packaging gears, water, plant materials, and other environmental resources with which humans and/or animals interact closely [49]. Further, the abundant presence of primary pathogens such as *Salmonella spp.* and *Shigella spp.* in HM is of great clinical significance because they have been implicated in deadly outbreaks of diarrheal diseases such as typhoid fever in some parts of Africa [8, 70]. According to the World Health Organization, such microbes should have zero (0%) presence in herbal medicine [71].

Overall, the least potent antibiotic drug was Ceftazidime; 95.10% (95% CI = 78.51–99.87%), followed by Ampicillin; 81.15 (95% CI = 47.61 to 99.21%), while the least resisted was Vancomycin (53.1%, n = 17). Our meta-analysis revealed that the least potent conventional drugs by bacteria isolated from HM in Africa are somewhat different from those that are highly resisted by bacterial contaminants of herbal products elsewhere. For example, in North America, resistance by bacteria such as *Bacillus spp.*, *Erwinia spp.*, *Staphylococcus spp.*, and *E. cloacae* isolated from commercial herbal products was reported in the order; Ampicillin, Nalidixic acid, Trimethoprim, Ceftriaxone, and Streptomycin [72]*.* As well, in Asia, the prevalence of resistance to various antibiotics by *S. aureus* herbal contaminants was in the order of; 93.33%, 90%, 86.66%, 70%, 63.33%, and 53.33% for penicillin, tetracycline, gentamicin, erythromycin, trimethoprim-sulfamethoxazole, and ciprofloxacin respectively [73]. The high resistance to Ceftazidime [a 3^rd^ generation cephalosporin (3GC) drug], in HM contaminants as reported in this meta-analysis, is in tandem with the recent reports of the worldwide surging incidence of 3GC-resistant gram-negative bacteria, mostly belonging to family Enterobacteriaceae [74]. Cephalosporins, more so the 3rd and 4th generation cephalosporins are listed by the World Health Organization among the critically important antimicrobial drugs for humans and animals, due to their remarkable efficacy against diarrheal pathogens such as *Salmonella spp*., *E. coli*, and *Campylobacter *spp., among others [75, 76]. The 3GCs are relatively safe antibiotics, that are extraordinarily active against enteric gram-negative bacilli and other critical pathogens such as *Haemophilus influenzae*, *Streptococcus agalactiae*, *Neisseria meningititidis*, and *Streptococcus pneumoniae* among others [77–79]. Third-generation cephalosporins, therefore, serve as first-line drugs in the management of major diseases such as; pneumonia, meningitis, urinary tract infections, sepsis, gonorrhea, skin and soft tissue infections among others [80]. Of recent, there has been a rapid emergence of 3^rd^ generation cephalosporin-resistant bacteria worldwide, including in Africa [81–83]. This raises worries related not only to the treatment-cost burdens but also the clinical implications [77].

The pooled prevalence of Extended Spectrum *β*-lactamase (ESBL) genes was 37.2%, and they were more predominant in some of the bacteria that possess potential significance in human medicine, such as; *Salmonella spp.* and *Proteus penneri*. In a study by Korir et al. 2017 in Kenya, specific ESBL types were examined; the *bla*_*CTX-M*_ and *bla*_*CMY*_ genes were detected at a prevalence of 34.4% and 32.3% respectively [14]. These findings are in contrast with the resistance genes recently identified in bacteria such as *S. aureus* that contaminated HM in some parts of Asia. In the latter, mostly Fluoroquinolone resistance genes were detected viz; *blaZ* (63.33%), *tetK* (60%), *ermA* (46.66%), *msrA* (43.33%), *aacA-D* (43.33%), and *mecA* (43.33%), *msrB* (6.66%), *ermB* (10%), *vanB* (13.33%), *fexA* (13.33%), *rpoB* (20%), and *vatB* (20%) [73]. The occurrence of ESBL in the bacterial contaminants of HM in Africa is of great concern because infections caused by these bacteria are very common in most parts of this continent [22, 47, 74, 84–86]. The ESBL genes code for ESBL enzymes that confer resistance to almost all *β*-lactam antibiotics (such as penicillin derivatives, cephalosporins, and monobactams). Most of these antibiotics target broad spectra of bacterial pathogens [74]. In recent years, the treatment challenges attributed to ESBL have become a global public health threat, because the strains that produce these enzymes constitute some of the most common MDR groups of medically important bacteria around the world [74].

This meta-analysis revealed that 5.8% (n = 6) of the bacterial contaminants screened, harbored plasmids of varying molecular weights, and these were suggested to potentially underpin the resistance phenotypes observed in the isolates in which they were detected. The presence of plasmids in the bacteria isolated from HM raises worries because such mobile genetic elements permit the rapid spread of non-chromosomal drug-resistance traits from one bacteria to another through horizontal gene transmission [87]. Such scientific phenomena could partly explain why antibiotic-susceptible strains of some bacteria, for instance, *S. aureus* and *E. coli* were reported to develop resistance to conventional antibacterial drugs when subjected to commercial herbal drug concoctions in the United States of America [72, 88]. Therefore, the escalation of antibacterial drug resistance that is potentially mediated by consumption of unhygienic herbal products is not exclusive to the low developed African states alone. This meta-analysis was limited by; online availability and the low number of research articles published on drug-resistant bacterial contamination of HM in Africa, the small number of countries that contained eligible studies, and the language (only English studies were available online among the eligible studies). In addition, some of the studies aimed at isolating a few target pathogens, and the isolates were tested against a limited number of antibiotics. Moreover, some researchers screened gram-negative bacteria against penicillin, yet such organisms are inherently resistant to this drug given their cell wall composition and structure [89].

## Conclusions

Herbal medicines in Africa possess highly drug-resistant bacterial contaminants. This points to a possible treatment failure when these contaminants are involved in diseases causation. More research on this subject should be done in the rest of the African countries, to fill the evidence gaps and support the formation of collaborative quality control mechanisms for the herbal medicine industry in Africa.

## Data Availability

Datasets generated and analyzed during this meta-analysis are available from the corresponding author on request.

## Abbreviations

AMR: Antimicrobial Resistance
ABR: Antibiotic Resistance
ESBL: Extended Spectrum β-lactamases
HM: Herbal Medicine
PRISMA: Preferred Reporting Items for Systematic Reviews and Meta-analyses
